# Risk factors for lymph node metastasis in gastric neuroendocrine tumor: a retrospective study

**DOI:** 10.1186/s12893-021-01174-7

**Published:** 2021-03-31

**Authors:** Xianghui Li, Lihua Shao, Xiaofeng Lu, Zhengyang Yang, Shichao Ai, Feng Sun, Meng Wang, Wenxian Guan, Song Liu

**Affiliations:** 1 Department of Gastrointestinal Surgery, Nanjing Drum Tower Hospital, The Affiliated Hospital of Nanjing University Medical School, 321 Zhongshan RD, Nanjing, 210008 China; 2 Department of General Surgery, Beijing Friendship Hospital, Capital Medical University, Beijing, 100050 China

**Keywords:** Gastric neuroendocrine tumor, Lymph node metastasis, Risk factors

## Abstract

**Background:**

Lymph node metastasis (LNM) plays a vital role in the determination of clinical outcomes in patients with gastric neuroendocrine tumor (G-NET). Preoperative identification of LNM is helpful for intraoperative lymphadenectomy. This study aims to investigate risk factors for LNM in patients with G-NET.

**Methods:**

We performed a retrospective study involving 37 patients in non-LNM group and 82 patients in LNM group. Data of demographics, preoperative lab results, clinical–pathological results, surgical management, and postoperative situation were compared between groups. Significant parameters were subsequently entered into logistic regression for further analysis.

**Results:**

Patients in LNM group exhibited older age (p = 0.011), lower preoperative albumin (ALB) (p = 0.003), higher carcinoembryonic antigen (CEA) (p = 0.020), higher International normalized ratio (p = 0.034), longer thrombin time (p = 0.018), different tumor location (p = 0.005), higher chromogranin A positive rate (p = 0.045), and higher Ki-67 expression level (p = 0.002). Logistic regression revealed ALB (p = 0.043), CEA (p = 0.032), tumor location (p = 0.013) and Ki-67 (p = 0.041) were independent risk factors for LNM in G-NET patients.

**Conclusions:**

ALB, CEA, tumor location, and Ki-67 expression level correlate with the risk of LNM in patients with G-NET.

## Background

Gastric neuroendocrine tumors (G-NET) formed by heterogeneous neoplasms arising from secretory cells of diffuse neuroendocrine system in stomach, one of the most common pathogenic site. Though G-NET is rare with a 1 ~ 2/1,000,000 incidence which accounts for 6.9 ~ 8.7% of all digestive neuroendocrine neoplasms per year, its incidence is increasing during recent decades worldwide [1–4]. Surgery serves as the first-line strategy for the management of G-NET [5]. Lymphadenectomy is required in patients with lymph node metastasis (LNM) for the prevention of recurrence and metastasis. Moreover, the rate of LNM in G-NET is higher than that in gastric adenocarcinoma [6]. Therefore, preoperative risk evaluation, diagnosis, and management of LNM in G-NET have become challenging issues.

World Health Organization (WHO) revised G grade in neuroendocrine tumors in 2010, in which G stands for grading according to mitotic count and Ki-67 index. The classifications and site-specific staging systems are mainly based on clinical pathology and immunohistochemistry, which provides limited information on LNM. Evidence regarding the preoperative identification of LNM in neuroendocrine tumors is very limited in current literature. In this circumstance, we aim to explore independent risk factors for LNM in patients with G-NET.

## Methods

### Patient selection

Between 2012 and 2019, all patients with G-NET that registered in Gastrointestinal Surgery of our hospital were recruited for qualification screening. The inclusion criteria were as follows: (1) definitive pathological diagnosis of G-NET; (2) the availability of pathological report; (3) absence of preoperative treatment including chemotherapy and radiotherapy. All patients in this study received D2 lymphectomy.

### Data collection

Data including demographics, preoperative lab results, clinical-pathological results, surgical management, and postoperative outcome were retrieved from the Electronic Medical Record System. Demographics included sex, age, underlying disease, past abdominal surgical history, and chief complaint. Preoperative lab result consisted of white blood cell count, neutrophil cell count, lymphocyte count, monocyte count, hemoglobin, platelet, albumin (ALB), C-reactive protein (CRP) levels, neutrophil-to-lymphocyte ratio (NLR), platelet-to-lymphocyte ratio (PLR), Onodera prognostic nutrition index (OPNI), fecal occult blood test, carcinoembryonic antigen (CEA), alpha-fetoprotein (AFP), CA125, CA199, CA242, CA724, international normalized ratio (INR), activated partial thromboplastin time (APTT), prothrombin time (PT) and thrombin time (TT). All these results were recorded from the last test before surgery. Clinical pathological results included lymph node metastasis, total number of resected lymph nodes, tumor size, tumor location, CD56, tumor proliferation index (Ki67), synaptophysin (Syn), and Chromogranin A (CgA). Surgical management included type of surgical procedure, duration of operation and intraoperative hemorrhage. Postoperative outcomes consisted of postoperative hospitalization time, postoperative oral feeding time and postoperative complications. All enrolled patients were divided into LNM group or non-LNM group according to the postoperative pathological report.

### Statistical analysis

All analyses were 2-tailed. The confidence interval was 5 ~ 95%, and p-values < 0.05 were defined as statistically significant. For continuous variables, data were presented as the mean ± SD (standard deviation), and unpaired t-test with Welch’s correction was applied for statistical analysis. For categorical variables, data were presented as frequency (percentage), and Chi-square test with Fisher’s exact test was conducted for statistical analysis. Significant variables in univariate analysis were brought into binary logistic regression model for multivariate analysis. And predicted risk factors were brought into receiver operation characteristic (ROC) curve analysis. All statistical analyses were performed in SPSS software (version 23.0; IBM Inc., Chicago, IL) and MedCalc software (version 11.4.2; MedCalc Software, Ostend, Belgium).

## Results

Between 2012 and 2019, a total of 122 G-NET patients that received surgery in our department were enrolled. According to the pathological report, 37 patients formed the non-LNM group, 82 patients formed the LNM group, and the other 3 patients were excluded due to the unavailability of lymph node information in pathological reports.

Table 1 compared demographics between two groups. Patients in LNM group were older than those in LNM group (p = 0.011). However, the difference in gender, hypertension, diabetes, other underlying diseases, past abdominal surgical history and chief complaint between two groups were not significant.

**Table 1:** Demographics of patients between non-LNM and LNM groups

	Non-LNM (n = 37)	LNM (n = 82)	P-value
Male (n, %)	24 (64.9%)	60 (83.2%)	0.357
Age (median ± SD)	61.22 ± 10.27	66.09 ± 9.16	0.011^*^
Hypertension (n, %)	11 (31.4%)	23 (28.8%)	0.772
Diabetes (n, %)	2 (5.7%)	8 (10.0%)	0.696
Other background disease (n, %)	2 (5.9%)	6 (7.7%)	0.732
Past abdominal surgery (n, %)	8 (22.9%)	21 (26.2%)	0.700
Chief complaint			0.410
Health examination	2 (5.7%)	4 (5.0%)	–
Pain	20 (57.1%)	35 (43.8%)	–
Melena/ hematemesis	4 (11.4%)	13 (16.2%)	–
Abdominal discomfort	9 (25.7%)	22 (27.5%)	–

^*^The asterisk indicates statistical significance

As showed in Table 2, higher ALB was observed in non-LNM group (p = 0.003), whereas higher INR (p = 0.034), longer TT (p = 0.018) and higher CEA (p = 0.020) were observed in LNM group. The differences of routine preoperative blood test, tumor biomarkers and other lab tests were not significant between groups. Especially, G grade was not significantly related to LNM.

**Table 2:** Preoperative lab test between patients in non-LNM and LNM groups

	Non-LNM (n = 37)	LNM (n = 82)	P-value
WBC (× 10^9^/L)	5.64 ± 1.55	6.35 ± 2.38	0.097
Neutrophils (× 10^9^/L)	3.53 ± 1.24	3.92 ± 2.13	0.307
Lymphocytes (× 10^9^/L)	1.56 ± 0.50	1.52 ± 0.61	0.749
Monocytes (× 10^9^/L)	0.69 ± 1.39	0.52 ± 0.75	0.412
Hb (g/L)	121.43 ± 26.31	114.01 ± 25.61	0.150
PLT (× 10^9^/L)	236.84 ± 85.12	228.91 ± 87.55	0.646
ALB (g/L)	39.93 ± 4.71	36.63 ± 5.69	0.003^*^
CRP (mg/L)	8.18 ± 14.88	16.70 ± 32.75	0.134
NLR	2.41 ± 0.88	3.00 ± 2.81	0.226
PLR	159.63 ± 58.80	165.78 ± 101.28	0.739
OPNI	46.03 ± 9.78	43.54 ± 8.54	0.166
Fecal occult blood test (n, %)			0.428
Negative	28 (75.7%)	56 (68.3%)	–
Positive	4 (10.8%)	13 (15.9%)	–
Unknown	5 (13.5%)	13 (15.9%)	–
AFP (ng/ml)	9.90 ± 27.94	5.30 ± 18.41	0.320
CEA (ng/ml)	2.41 ± 3.29	12.25 ± 23.78	0.020^*^
CA125 (U/ml)	23.84 ± 68.17	13.05 ± 15.03	0.203
CA199 (U/ml)	9.29 ± 7.14	27.82 ± 96.43	0.274
CA242 (U/ml)	4.05 ± 3.16	6.60 ± 9.92	0.213
CA724 (U/ml)	2.18 ± 3.56	6.88 ± 23.36	0.312
INR	1.01 ± 0.77	1.04 ± 0.78	0.034^*^
APPT (s)	26.41 ± 5.10	28.25 ± 5.30	0.081
PT (s)	17.66 ± 3.31	18.32 ± 1.85	0.171
TT (s)	11.53 ± 0.87	11.96 ± 0.94	0.018^*^

^*^The asterisk indicates statistical significance

The distribution of tumor location was different between non-LNM and LNM groups (p = 0.005), although the tumor in both groups preferred cardia and fundus of stomach. Besides, higher Ki-67 index (p = 0.002) and higher CgA positive rate (p = 0.045) were found in LNM group (Table 3).

**Table 3:** Tumor characteristics of patients between non-LNM and LNM groups

	Non-LNM (n = 37)	LNM (n = 82)	P-value
Tumor size (cm)	4.44 ± 3.81	5.37 ± 2.54	0.120
Tumor size classification (n, %)			0.062
< 5 cm	27 (73.0%)	45 (54.9%)	–
≥ 5 cm	10 (27.0%)	37 (45.1%)	–
G grade (n, %)			0.210
G1	3 (8.3%)	5 (6.1%)	–
G2	0 (0.0%)	4 (4.88%)	–
G3	22 (61.1%)	59 (72.0%)	–
NEC	11 (30.6%)	14 (17.1%)	–
Tumor location (n, %)			0.005^*^
Cardia and fundus of stomach	16 (43.2%)	46 (56.3%)	–
Body of stomach	9 (24.3%)	30 (36.6%)	–
Pyloric antrum	7 (18.9%)	5 (6.5%)	–
Pyloric canal	5 (13.5%)	1 (1.2%)	–
CD56 (n, %)			0.177
–	9 (27.3%)	15 (21.9%)	–
+	12 (36.4%)	32 (46.4%)	–
++	8 (24.2%)	7 (10.1%)	–
+++	4 (12.1%)	15 (21.7%)	–
Unknown	4 (12.1%)	13 (15.9%)	–
Ki67	43.3 ± 27.6%	58.3 ± 21.6%	0.002^*^
Syn (n, %)			0.405
–	0	1 (1.2%)	–
+	14 (37.8%)	22 (26.8%)	–
++	5 (13.5%)	20 (24.4%)	–
+++	17 (45.9%)	37 (45.1%)	–
Unknown	1 (2.7%)	2 (2.4%)	–
CgA (n, %)			0.045^*^
–	9 (24.4%)	22 (26.8%)	–
+	14 (37.8%)	31 (37.8%)	–
++	0	11 (13.4%)	–
+++	13 (35.1%)	15 (18.3%)	–
Unknown	1 (2.7%)	3 (3.7%)	–

^*^The asterisk indicates statistical significance

There was no statistical difference in surgical procedure, duration of operation, intraoperative hemorrhage, postoperative hospitalization, postoperative oral feeding and postoperative complications between two groups (Table 4). A certain proportion of patients were transferred into ICU after surgery (38.5% and 32.9% in two groups, respectively). The ICU stay is mostly 1 day for patients in both groups (Table 4).

**Table 4:** Surgical procedures and outcome between patients in non-LNM and LNM groups

	Non-LNM (n = 37)	LNM (n = 82)	P-value
Surgical procedure			0.119
Local resection	2 (5.4%)	1 (1.2%)	–
Distal gastrectomy	9 (24.3%)	9 (11.0%)	–
Proximal gastrectomy	6 (16.2%)	13 (15.9%)	–
Total gastrectomy	20 (54.1%)	59 (72.6%)	–
Duration of operation (min)	225.97 ± 84.84	239.83 ± 71.07	0.367
Intraoperative hemorrhage (ml)	338.57 ± 541.93	260.13 ± 259.23	0.303
Postoperative hospitalization (d)	14.97 ± 10.16	13.49 ± 5.44	0.311
Postoperative oral feeding (d)	9.09 ± 6.46	7.61 ± 2.91	0.093
Postoperative complications (n, %)	1 (2.7%)	1 (1.2%)	0.509
ICU stay (n, %)	15 (38.5%)	27 (32.9%)	0.376

Binary logistic regression was further conducted. Significant variables identified in previous univariate analysis (including age, ALB, CEA, INR, TT, tumor location, Ki67 and CgA) were enrolled into the regression model. ALB (p = 0.004), CEA (p < 0.001), tumor location (p = 0.006) and Ki67 (p = 0.041) were statistically significant between non-LNM and LNM groups (Table 5).

**Table 5:** Logistic regression analysis of risk factors for lymph node metastasis in G-NET

	OR	95% CI	P-value
ALB	0.887	0.789–0.996	0.043^*^
CEA	1.113	1.009–1.228	0.032^*^
Tumor location			0.013^*^
Cardia and fundus of stomach^#^	–	–	–
Body of stomach	6.920	0.585–81.856	0.125
Pyloric antrum	10.733	0.840–137.107	0.068
Pyloric canal	0.238	0.008–7.373	0.413
Ki-67	8.174	1.085–61.568	0.041^*^

^*^The asterisk indicates statistical significance

^#^“cardia and fundus of stomach” was assigned as the reference in logistic regression analysis

**Fig. 1:**
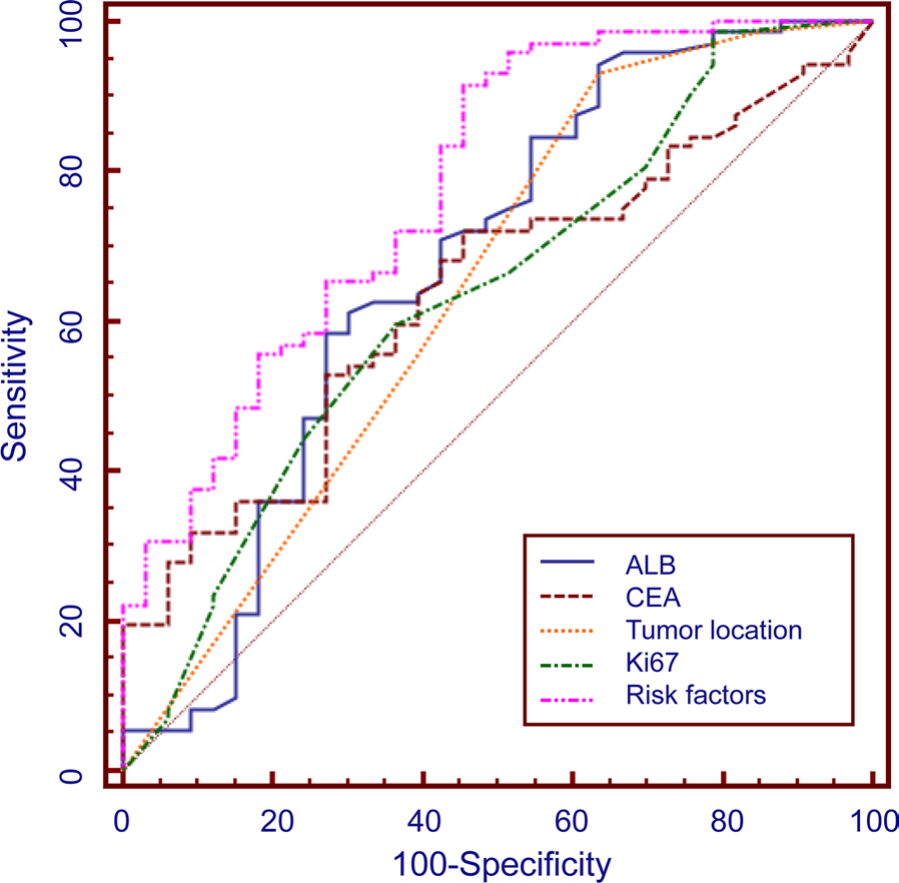
ROC curve for risk factors of lymph node metastasis in G-NET. The ROC curve of ALB, CEA, Ki67, tumor site, and the integrated diagnostics to lymph node metastasis. (ALB: AUC = 0.707, 95% CI = 0.616 ~ 0.787, p = 0.0002; CEA: AUC = 0.642, 95% CI = 0.543 ~ 0.733, p = 0.0101; tumor location: AUC = 0.618, 95% CI = 0.524 ~ 0.705, p = 0.0344; Ki67: AUC = 0.657, 95% CI = 0.564 ~ 0.742, p = 0.0044; Integrated risk factors: AUC = 0.779, 95% CI = 0.688 ~ 0.855, p < 0.0001)

**Fig. 2:**
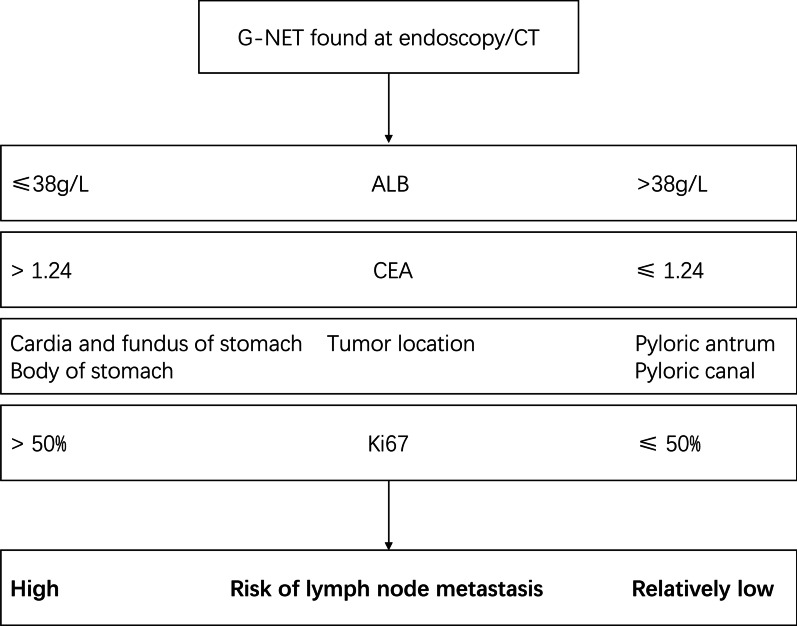
Proposed approach for the risk evaluation of lymph node metastasis in G-NET. The cut-off values were extracted from previous ROC curve analysis

Subsequent ROC analysis calculated the diagnostic value of each risk factor. The AUC area for ALB, CEA, tumor location and Ki-67 was 0.707, 0.642, 0.618 and 0.657, respectively. Integration of all risk factors exerts a better diagnostic capacity (AUC = 0.779, 95% CI = 0.688 ~ 0.855, p < 0.0001) (Fig. 1). These data generated a proposed approach for the risk evaluation of lymph node metastasis in G-NET (Fig. 2).

## Discussion

Here is a summary of our main findings. G-NET is rare and LNM plays an immense role in the prognosis [7]. However, current guidelines fail to provide relevant recommendation to the risk evaluation and management of LNM. European Neuroendocrine Tumor Society (ENETS) guidelines didn’t provide relevant information in the 2012 version [8]. In the 2016 updated version, surgical treatment is recommended as the first-line strategy that follows strategies employed for gastric adenocarcinomas (partial or total gastrectomy with lymph node dissection) [9]. Similar recommendation was provided by the North American Neuroendocrine Tumor Society (NANETS) [10, 11]. In the 2017 NANETS guideline, metastatic G-NET are recommended to be treated in a similar fashion as other malignant carcinoids. National Comprehensive Cancer Network (NCCN) guidelines suggested that resection of gastrointestinal neuroendocrine tumors should include adequate regional lymph node resection [12]. However, in what situation lymph node resection is necessary and what kind of lymphectomy should be performed were not discussed. Chinese Clinical Oncology guidelines propounded that lymph node resection should be conducted for patients with distant metastasis, LNM or diagnosed as G3 grade [13]. In summary, current guidelines could not provide enough information for the management of G-NET with LNM. Moreover, it was difficult to fully exclude LNM before surgery.

By comparing G-NET patients with or without LNM, we found that older age, preoperative lower albumin level, higher CEA level, higher INR, longer TT, higher Ki67, and CgA positive rate were associated with lymph node metastasis. Logistic regression identified that ALB, CEA, tumor location and Ki67 were independent risk factors for LNM in patients with G-NET. There has been an increasing incidence of G-NET in recent decades [14]. Surgical resection is the first-line recommendation for G-NET [15]. However, it is largely unknown how to determine the possibility of LNM preoperatively. Here, our study has provided useful information that ALB, CEA and Ki67 as well as tumor location are associated with the risk of LNM in G-NET.

As with other digestive NETs, our patients were divided into several groups according to the WHO G grade classification. Albeit G grade has exerted the versatile negative prognostic factor in digestive NETs from pancreas and jejunum-ileum, its diagnosis value for determining the prognosis of patients with G-NET didn’t live up to expectation [16]. Likewise, the lack of solid evidence situated on the G grade effective on G-NET lymph node metastasis aroused our interest. However, resultant data retard the harnessing of G grade system to predict nodal metastasis. We sought to figure out risk factors with forecasted usage value to address this issue in this scenario.

Serum ALB level is an easily accessible laboratory indicator that reflects individual nutritional status. Previous study has demonstrated that albumin is a vital source of energy and amino acids for tumor cells, and it was increasingly absorbed by tumor cells owing to fast growth and active metabolism of tumors [17]. In addition, ALB is considered as an indicator of systemic inflammatory reaction in malignant tumors. G-NET potentially affects digestive and absorptive ability and is associated with systemic inflammatory response. Both reasons lead to suppressed synthesis of ALB, which was found more severe in G-NET patients with LNM [18]. A study involving 207 patients with malignant tumors reported that patients with lower prognostic nutrition index exhibited higher lymph node metastasis rate. Another retrospective study recruited 136 patients found that lymph node invasion was significantly correlated with ALB level [19]. Moreover, a scoring system named Glasgow prognostic score based on inflammation (CRP and ALB) has been validated as versatile in predicting progress for gastric cancer [20].

CEA is associated with various types of cancer including gastric cancer and correlated with overall survival of patients [21, 22]. A study in China found that increased CEA levels were associated with LNM in remnant gastric cancer [23]. Another study discovered that gastroenteropancreatic neuroendocrine neoplasm patients with elevated CEA, CA125 or CA19-9 exhibited worse overall survival [24]. Nevertheless, there were littele data about the relationship between CEA and LNM in NET. Our study found that elevated CEA could serve as a predicting factor of LNM in G-NET.

There were few studies discussing the correlation between tumor location and LNM in G-NET. Liang J et al. revealed that G-NET is mainly located in esophagogastric junction, most of which were aggressive malignant [25]. To our knowledge, our findings are the first investigation towards tumor location and LNM in G-NET. Our study has highlighted the tumor distribution in stomach associated with LNM manifestation in this specific cohort of patients. As for clinicians, LNM is worthy of more concern facing the G-NET patient whose tumor is located in cardia and fundus of stomach and body of stomach.

The nuclear antigen Ki67 structurally associated with chromatin helps determine tumor grade and prognosis [26]. Previous studies suggested a significant correlation between Ki-67 level and clinical outcome. Boo et al. revealed that higher Ki67 (> 60%) was associated with aggressive G-NET [27]. Another study illustrated that higher Ki67 was not only associated with higher T stage (p = 0.003) but also tended to be associated with LNM (p = 0.071) [28]. In accordance with previous reports, our study revealed that higher Ki67 could serve as an independent predictive factor for LNM in G-NET. When it comes to neuroendocrine tumors, Ki67 is the major prognostic factor and utilized in the novel grading system [29].

We are aware of potential limitations. First, this is a single-center retrospective study that may lead to selection bias. Second, since the short-term outcome between two groups was not significant and follow-up data is not fully available, we could not compare long-term outcomes of G-NET patients with or without LNM. Third, the rarity of G-NET and limited sample size hampers subgroup analysis such as distant metastasis compared to adjacent metastasis. In addition, molecular analysis was conducted in very few patients, which leads to failure of comparison of molecular features between LNM and non-LNM groups. Nevertheless, our study has provided a comprehensive exploration towards possible risk factors of LNM in G-NET. Future prospective studies are expected to provide more information for the identification of LNM in G-NET.

## Conclusion

In conclusion, ALB, CEA, tumor location and Ki67 correlate with the risk of LNM in patients with G-NET.

## Data Availability

We declared that materials described in the manuscript, including all relevant raw data, will be freely available to any scientist wishing to use them for non-commercial purposes. The datasets used and analyzed during the current study are available from the corresponding author upon reasonable request without breaching participant confidentiality.

## Abbreviations

LNM: Lymph node metastasis
G-NET: Gastric neuroendocrine tumor
WHO: World Health Organization
ALB: Preoperative albumin
CEA: Higher carcinoembryonic antigen
INR: International normalized ratio
TT: Thrombin time
Ki67: Tumor proliferation index
CgA: Chromogranin A
ROC: Receiver operation characteristic
